# Assessment of Grassland Carrying Capacity and Grass–Livestock Balance in the Three River Headwaters Region Under Different Scenarios

**DOI:** 10.3390/biology14080978

**Published:** 2025-08-01

**Authors:** Wenjing Li, Qiong Luo, Zhe Chen, Yanlin Liu, Zhouyuan Li, Wenying Wang

**Affiliations:** 1 College of Geography Science, Qinghai Normal University, Xining 810008, China; lwenjingjing@163.com; 2 College of Life Sciences, Qinghai Normal University, Xining 810008, China; 3 School of Grassland Science, Beijing Forestry University, Beijing 100083, China; 4 Qinghai Provincial People’s Government-Beijing Normal University Institute of Plateau Science and Sustainable Development, Xining 810008, China

**Keywords:** different scenarios, grassland carrying capacity, grass–livestock balance, artificial supplementary feeding, crude protein, sustainable development, grassland management

## Abstract

It is crucial to clarify the grassland carrying capacity (CC) and the balance between grass and livestock under different scenarios for ecological protection and sustainable development in the Three River Headwaters Region (TRHR). This study focused on the TRHR and used livestock data, MODIS Net Primary Productivity (NPP) data, and artificial supplementary feeding data to analyze grassland CC and explore changes in the grass–livestock balance across various scenarios. The results showed that the theoretical CC of edible forage under complete grazing conditions was much lower than that of crude protein under nutritional carrying conditions. Furthermore, without increasing the grazing intensity of natural grasslands, artificial supplementary feeding reduced overstocking areas by 21%. These results suggest that supplementary feeding effectively addresses the imbalance between forage supply and demand, serving as a key measure for achieving sustainable grassland livestock husbandry. Despite the effective mitigation of grassland degradation in the TRHR due to strict grass–livestock balance policies and ecological restoration projects, the actual livestock CC exceeded the theoretical capacity, leading to overgrazing in some areas. To achieve desired objectives, more effective grassland management strategies must be implemented in the future to minimize spatiotemporal conflicts between grasses and livestock and ensure the health and stability of grassland ecosystems.

## Simple Summary

The carrying capacity (CC) of grasslands, as well as the balance between grass and livestock in areas where only the natural supply of pasture is considered, has been extensively studied. However, these studies do not fully or accurately reflect the CC of grasslands. Therefore, we analyzed changes in grassland CC and grass–livestock balance in the Three River Headwaters Region (TRHR) under different scenarios based on livestock, MODIS Net Primary Productivity (NPP), and artificial supplementary feeding data. The results showed that the theoretical CC of crude protein in edible forage is high and that artificial supplementary feeding can effectively alleviate overgrazing. Although ecological restoration projects have increased grass yield, sustainable grassland management strategies, such as artificial supplementary feeding and advanced grassland management measures, are crucial for minimizing conflicts between grass and livestock. These research findings are significant for promoting the coordinated economic and ecological development of the TRHR, ensuring the sustainable development of grasslands, and safeguarding regional well-being.

## 1. Introduction

Grasslands are one of the most widespread types of vegetation globally. They cover approximately 31.5% of the Earth’s land area and 41.7% of China’s land area [1,2,3]. Grasslands play a vital role in carbon cycling, water conservation, biodiversity protection, and climate regulation. Furthermore, grasslands serve as the foundation for livestock husbandry development, supporting agricultural progress and sustaining the livelihoods of pastoral communities, while significantly influencing regional economic development [4,5]. However, studies have suggested that moderate grazing (of light-to-moderate intensity) could help reduce soil erosion, enhance grassland productivity, and maintain biodiversity. Conversely, prolonged heavy grazing often led to reduced vegetation cover and biomass, as well as degradation of soil physical, chemical, and biological properties, which in turn caused a gradual decline in grassland productivity and varying degrees of grassland degradation. This exacerbated the conflict between livestock production and ecological conservation [6,7,8]. Therefore, achieving an appropriate balance between grass and livestock is essential for preserving natural grassland functions and ecological equilibrium, as well as ensuring regional ecological protection and the sustainable development of livestock husbandry.

The Three River Headwaters Region (TRHR) is the source of the Yangtze River, the Yellow River, and the Lancang River. It serves as an important water conservation area and ecological security barrier in China, as well as being the region with the highest concentration of plateau biodiversity in the country [9]. Grasslands are the dominant ecosystem type in this region, accounting for approximately 71.2% [10]. This establishes grasslands as a crucial foundation for livestock husbandry and indicates that the balance between grass and livestock directly impacts the degradation and restoration of grasslands. However, since the 1980s, the fragile alpine grassland ecosystem has faced severe degradation due to the interplay of various factors and unsustainable grassland use. This has led to increasingly prominent conflicts between grasslands and livestock [4,11], posing significant challenges to the sustainable development of regional grassland livestock husbandry and the area’s ecological status. Research has indicated that overgrazing is one of the key factors contributing to grassland degradation and decreased productivity, posing a significant threat [12].

Grassland carrying capacity (CC) is one of the important concepts in pasture management [13], and a key indicator for developing the grassland livestock husbandry. It is also an important basis for implementing a system that determines livestock based on grass and maintains a balance between grass and livestock [3]. Artificial supplementary feeding and the nutrient content of forage are crucial variables that influence the accuracy of calculating grassland CC [14,15]. As a significant regulatory measure in contemporary grassland management, artificial supplementary feeding effectively regulates the supply–demand relationship between grass and livestock by providing external forage. This practice alleviates seasonal pressure on grasslands and serves an irreplaceable buffering role, particularly during drought periods or in overgrazed areas. The crude protein content in forage is widely acknowledged as the “gold standard” for evaluating forage nutritional value, owing to its direct influence on livestock growth, reproduction, and production efficiency [16]. The levels of crude protein serve as a direct reflection of the nutritional supply capacity of pastures, facilitating precise calculations of CC, the regulation of grazing intensity, and providing guidance for effective high-quality pasture management [17]. However, most existing studies on grassland CC and the grass–livestock balance in the TRHR have primarily focused on localized areas or have only considered the supply of natural forage [18,19]. These studies frequently overlook the two key factors mentioned earlier. Consequently, relying solely on natural forage yield data may lead to incomplete and potentially biased assessments of grassland CC, which do not fully capture the overall dynamics of the region’s pastoral system. In summary, this study took a comprehensive approach to considering factors such as natural grassland conditions, artificial supplementary feeding, and crude protein yield. The objective was to improve the scientific rigor and accuracy of assessing the TRHR’s grassland CC. Additionally, the study aimed to provide basic data and guidance for decision-making regarding management practices in regional grasslands. Ultimately, we sought to promote the coordinated development of the regional grassland livestock husbandry. 

## 2. Materials and Methods

### 2.1. Study Area

**Figure 1:**
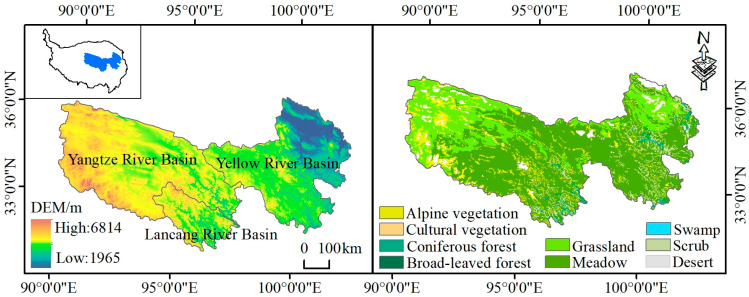
Study area and vegetation types.

The TRHR, located in the hinterland of the Tibetan Plateau at coordinates 31°39′–36°12′ N and 89°45′–102°23′ E, occupies 50.43% of the total area of Qinghai Province (Figure 1). The terrain is high in the west and low in the east, with an average elevation of over 4000 m. The region has a typical continental plateau climate, marked by significant temperature variations between day and night, long hours of sunshine, and intense radiation. It receives 2300 to 2900 h of sunshine per year, with an average temperature ranging from −5 to 4 °C and an annual precipitation ranging from 200 to 800 mm. Annual evapotranspiration ranges from 730 to 1700 mm [20,21]. Grasslands and meadows are the most widespread ecosystem types in the region, with significant differences in vegetation between the east and west.

Livestock husbandry is the main industry in this region. The eastern part serves as an agro-pastoral transitional zone, and the western part comprises the uninhabited Hoh Xil area. Some herders continue to practice seasonal migration, moving to the mountains in summer and to the valleys in winter. Studies have demonstrated that the TRHR can support approximately 1.356 million sheep units (SU), exhibiting a gradient pattern where the Lancang River region has the highest livestock carrying capacity, followed by the Yellow River source region, while the Yangtze River source region has the lowest. The pressure index stood at 1.65, which exceeded the CC by 65% [22]. However, following the implementation of livestock reduction measures in 2003, the total livestock population has decreased by 11.1% [23], resulting in a reduction of overgrazing in the TRHR.

### 2.2. Data Sources

Vegetation NPP data (MOD17A3HGF version) were obtained from the National Aeronautics and Space Administration (NASA, Washington, DC, USA) Data Center at a spatial resolution of 500 m. This dataset provides several significant advantages, including extensive geographical coverage, continuous time series data, rigorous accuracy validation, and reliability and comparability on a global scale [24,25]. We conducted a series of preprocessing steps using ArcGIS 10.8, which included projection conversion, pixel resampling to 1 km, and cropping of the study area data. Livestock data were obtained from the Qinghai Province Survey Team of the National Bureau of Statistics. Artificial supplementary feeding data were obtained from the Qinghai Provincial Grassland General Station.

### 2.3. Research Methods

#### 2.3.1. Grass Yield Calculation and Mann-Kendall (MK) Test

To calculate the equilibrium state of grass and livestock, the NPP must be converted to an edible hay amount. In the Qinghai region, the productivity of the underground part is approximately four times that of the aboveground part [26], while the carbon content of the aboveground part is 0.43 [27]. Therefore, the formula for calculating grass yield in the TRHR was derived from existing studies [20], and is as follows:

(1) G=NPP/2.15

where G is the aboveground hay weight per unit area g/(m^−2^⸱a^−1^).

Additionally, we employed the MK test to quantitatively analyze the spatio-temporal trends in grass yield within the TRHR from 2000 to 2023. We categorized these trends into nine distinct categories to identify the significant types of changes in grass yield in the TRHR.

#### 2.3.2. Calculation of Actual Livestock Carrying Capacity (CC)

We converted the counties’ livestock statistics into standard sheep units. Large livestock were calculated as four sheep units, while sheep and goats were each considered one sheep unit. Considering that Hoh Xil, located in the northwest of the TRHR, is an uninhabited area, we excluded it from our calculations and recorded the livestock count in this region as zero. The formula is as follows:

(2) $$N_{s}=\sum (M_{i}\times R_{i})/Area$$

where $N_{s}$ is the actual number of livestock unit; i is the type of livestock units; $M_{i}$ is the number of livestock; $R_{i}$ is the conversion coefficient of the standard livestock unit; and Area is the size of the grassland.

#### 2.3.3. Calculation of Theoretical Livestock Carrying Capacity (CC)

Theoretical CC is basically the max number of livestock that can graze on a certain area of grassland in moderate conditions. It is one of the main indicators used to measure grassland productivity and can be assessed based on forage growth conditions (including yield and quality). The calculation formula is [28]:

(3) $$N_{c}=(G\times K\times U)/(R\times 365)$$

In this formula, $N_{c}$ represents the theoretical CC (SU/hm^2^); G represents the grass yield per unit area (kg/hm^2^); K represents the proportion of edible forage in the grassland. This is calculated at 80%, based on related studies [29,30]; and U represents the reasonable utilization rate of the grassland. This is calculated at 50%. R represents the standard daily feed intake for one sheep, as determined by the current standard, NY/T 3647-2020 (https://www.codeofchina.com/standard/NYT3647-2020.html, accessed on 21 March 2025), which sets the standard at 1.6 kg/day.

#### 2.3.4. Calculation of Crude Protein Yield of Edible Forage

The crude protein yield was estimated using mean crude protein content values from the green-up (17.29%), peak growth (10.43%), and senescence (5.60%) periods in the TRHR, as estimated in an existing study [23]. The crude protein yield was calculated as follows:

(4) Y =G×K×U×P

where Y is the crude protein yield of grass, G, K, and U have the same meanings as before, and P is the percentage of crude protein content.

#### 2.3.5. Calculation of Grass and Livestock Balance and Overgrazing Rate in Different Scenarios

The grass–livestock balance and overgrazing rate is defined as the ratio of the realistic grass–livestock CC $N_{s}$ to theoretical CC $N_{c}$.

Under complete grazing conditions, it is calculated as follows [31]:

(5) $$B_{1}=(N_{s}-N_{c})/N_{c}$$

where $B_{1}$ is the grass–livestock balance and overgrazing rate.

After considering artificial supplementary feeding, the calculation formula is:

(6) $$B_{2}=\frac{N_{s}-\left(N_{c}+N_{b}\right)}{\left(N_{c}+N_{b}\right)}$$

$N_{b}$ represents the amount of supplementary artificial forage.

After considering forage nutrition, the formula is calculated as:

(7) $$B_{3}=(N_{s}-N_{Y})/N_{Y}$$

$N_{Y}$ represents the nutritional CC of edible forage. According to relevant studies [23,32], the daily requirement for digestible crude protein to maintain the basic metabolic needs of a standard sheep is 53.9 g. To sustain a standard weight of 45 kg for a sheep, the daily requirement of digestible crude protein is 94 g. For a growing standard sheep, the daily digestible crude protein requirement is 152 g per 100 g of growth.

#### 2.3.6. Evaluation Grade Classification of Grass–Livestock Balance and Overgrazing Rate

According to the current relevant standard LY/T 3322-2022 (https://www.gbstandards.org/China_industry_standard_english.asp?code=LY/T%203322-2022, accessed on 21 March 2025) and Qinghai Province local standard DB 63/T 2334-2024 (https://dbba.sacinfo.org.cn/stdDetail/6036c6f960c2d25148d0b9d69e048c0ea0cf683b30c25cbefe4f0f96ce91ffd5, accessed on 21 March 2025), the grass–livestock balance and CC are divided into four grades (Table 1): understocking (B < 0), balanced stocking (0 ≤ B < 15%), overstocking (15% ≤ B < 50%), and serious overstocking (B ≥ 50).

## 3. Results

### 3.1. Analysis of Grass Yield

**Figure 2:**
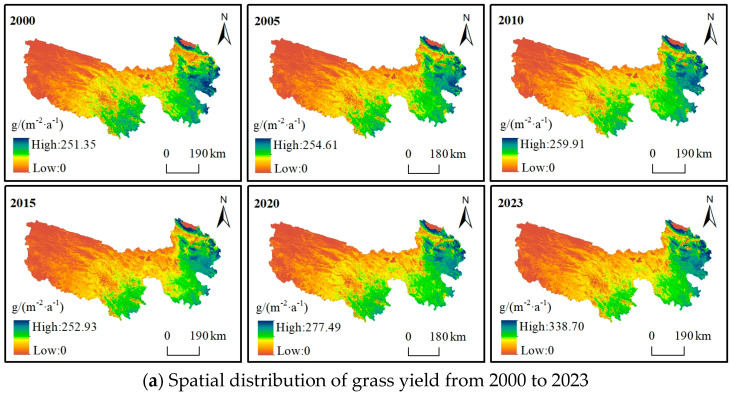

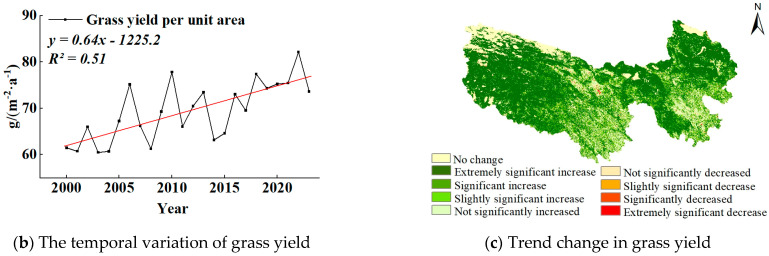
(**a**) Spatial distribution of grass yield from 2000 to 2023; (**b**) the temporal variation of grass yield; (**c**) trend change in grass yield.

From a spatial perspective (Figure 2a), grass yield (unit yield) has remained largely consistent from 2000 to 2023, gradually decreasing from the southeast to the northwest. In the northwest, grass yield ranges from 0 to 60 g/(m^−2^·a^−1^); in the central–south region, it ranges from 70 to 110 g/(m^−2^·a^−1^); and in the east, it ranges from 120 to 340 g/(m^−2^·a^−1^). This pattern aligns with the distribution of water and heat in the alpine region, as well as the spatial distribution of precipitation, temperature, and altitude. Over the past 24 years, the maximum grass yield per unit area in the TRHR has ranged from 231.40 to 338.70 g/(m^−2^·a^−1^). The highest yield, at 338.70 g/(m^−2^·a^−1^), was recorded in 2023. From a temporal perspective (Figure 2b), the average grass yield per unit area in the TRHR ranged from 60.51 to 82.18 g/(m^−2^·a^−1^) between 2000 and 2023, showing a fluctuating increase with an average increase of approximately 0.64 g/(m^−2^·a^−1^). According to trend analysis (Figure 2c), most regions within the TRHR exhibited an upward trend from 2000 to 2023. Areas with significant increases accounted for 83.26% of the total area, and areas with extremely significant increases exceeded 57.10%. Areas with significant decreases were sporadic, occurring only in southern Qumalai, southeastern Zhiduo, and the Gande and Jiuzhi regions.

### 3.2. Temporal and Spatial Distribution of Actual Livestock Carrying Capacity (CC)

**Figure 3:**
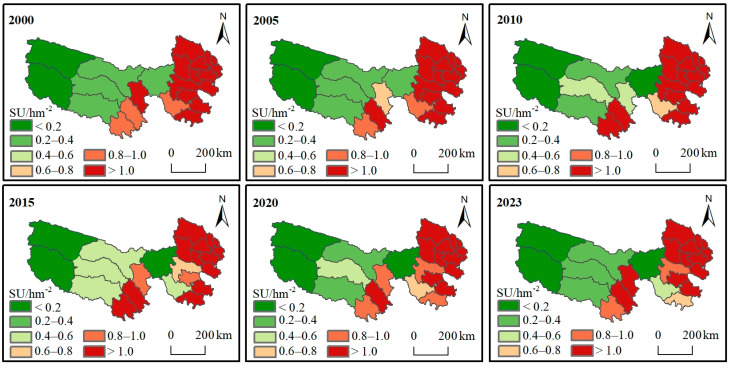
Distribution of actual livestock carrying capacity (CC).

The livestock in the counties and cities of the TRHR were uniformly converted into standard sheep units (SU). Then, the grassland area was calculated for each county and city. Finally, the actual livestock CC was calculated based on the number of livestock and the grassland area. In terms of spatial and temporal distribution (Figure 3), the actual livestock CC in the TRHR from 2000 to 2023 was highest in the east, second highest in the center, and lowest in the west. The actual livestock CC was smallest in the Hoh Xil forbidden grazing area and Tanggula town in the west, consistently remaining below 0.2 SU/hm^−2^. Conversely, Yushu and other eastern counties and cities had the highest actual CC, consistently exceeding 1.0 SU/hm^−2^. According to statistics, the region’s average actual CC was 0.7840 SU/hm^−2^. Overall, over the past 24 years, the actual CC in the TRHR has shown an increasing trend, with an annual growth rate of 0.0058 SU/hm^−2^.

### 3.3. Spatial and Temporal Distribution of Theoretical Carrying Capacity (CC)

#### 3.3.1. Theoretical Spatial and Temporal Distribution of Livestock Carrying Capacity (CC) Under Complete Grazing Conditions

**Figure 4:**
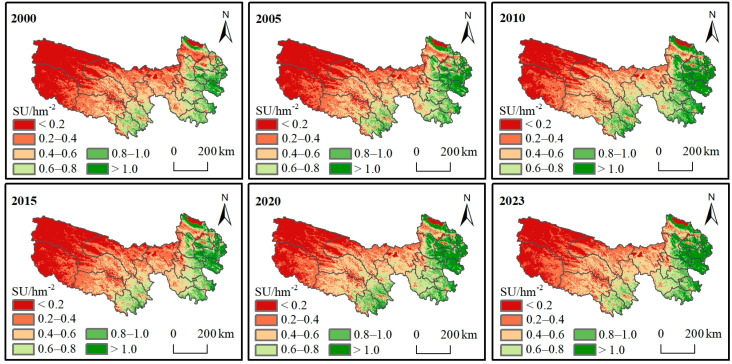
Distribution of theoretical carrying capacity (CC).

As shown in Figure 4, the distribution of the theoretical livestock CC was consistent with the grass yield distribution pattern, exhibiting the largest capacity in the southeast, followed by the center, and the smallest capacity in the northwest. The theoretical livestock CC exceeded 0.6 SU/hm^−2^ in the southeast, ranged from approximately 0.2 to 0.6 SU/hm^−2^ in the center, and remained below 0.2 SU/hm^−2^ in the northwest. The maximum multi-year average of the theoretical livestock CC in the TRHR reached 1.70 SU/hm^−2^, primarily concentrated in the eastern part of the region. Over the past 24 years, the regional mean theoretical CC of the TRHR has ranged from 0.37 to 0.50 SU/hm^−2^, with an average of 0.44 SU/hm^−2^. There has been an overall significant upward trend, with an annual increase of 0.0041 SU/hm^2^. However, the actual CC has increased by 0.0058 SU/hm^−2^ per year, which was 0.0017 SU/hm^−2^ more than the increase in theoretical CC.

#### 3.3.2. Theoretical Crude Protein Carrying Capacity (CC) of Edible Pasture Under Nutrient Loading

As shown in Table 2, the average crude protein yield of the TRHR during the period from 2000 to 2023 ranged from 2.54 to 3.21 g/m^−2^, with an average yield of 2.89 g/m^−2^. This was calculated based on the crude protein yields in different periods and the theoretical CC of crude protein under various nutritional demands. The average theoretical CC of crude protein varied depending on the nutritional demands: 14.68 SU/hm^−2^ for basic metabolic requirements, 8.42 SU/hm^−2^ for maintaining a body weight of 45 kg, and 5.21 SU/hm^−2^ for a daily weight gain of 100 g. Overall, the theoretical CC of edible forage under complete grazing conditions was significantly lower than that of crude protein under specific nutritional demands. Additionally, the theoretical CC of crude protein in edible forage varied significantly depending on the level of crude protein required.

This result is consistent with existing studies. He et al. [23] previously estimated the crude protein yields for the alpine grassland of the TRHR: 4.98 SU/hm^−2^ during the regreening period, 8.30 SU/hm^−2^ during the peak growth period, 0.89 SU/hm^−2^ during the withering period, and 14.17 SU/hm^−2^ annually. To maintain a body weight of 45 kg, the values were 2.85 SU/hm^−2^ during the regreening period, 4.76 SU/hm^−2^ during the peak growth period, 0.51 SU/hm^2^ during the withering period, and 8.12 SU/hm^−2^ for the entire year.

### 3.4. Grass–Livestock Balance Under Different Scenarios

#### 3.4.1. The Grass–Livestock Balance Under Complete Grazing Conditions

**Figure 5:**
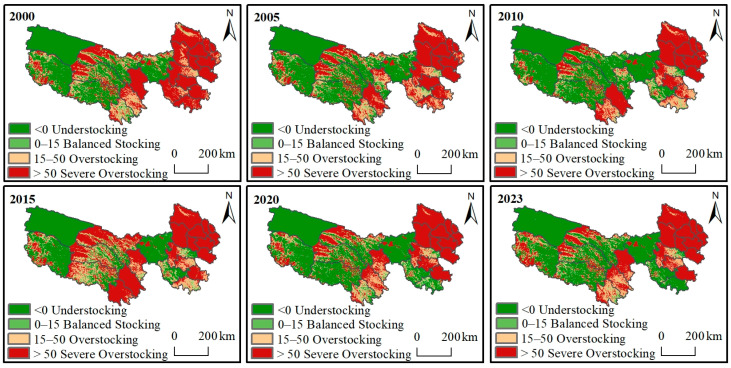
Spatial distribution of grass and livestock balance under complete grazing conditions.

As shown in Figure 5, the spatial distribution of grassland grazing pressure in the TRHR under complete grazing conditions from 2000 to 2023 showed a gradual aggravation trend from west to east. Overall, the areas with understocking and severe overstocking were more extensive, while those with balanced stocking and overstocking were scattered. Specifically, 41.3% of the region was understocking, 6.7% was balanced stocking, 12.7% was overstocking, and 39.3% was severe overstocking. Under complete grazing conditions, 52% of the TRHR was overstocked, while 48% was not.

#### 3.4.2. The Grass–Livestock Balance Under Artificial Supplementary Feeding Conditions

In addition to consuming natural pastures, livestock primarily depend on supplementary feeds such as straw, grains, and silage. In the TRHR, supplementary forage crops are predominantly *Avena sativa*, *Zea mays*, *Elymus dahuricus*, *Festuca sinensis*, *Lolium perenne*, and *Medicago sativa*. According to the data on artificial supplementary forage provided by the Qinghai Provincial Grassland General Station for the TRHR, the artificial supplementary feeding situation varies significantly across different county areas, with an annual total hay production of 387,000 tons.

**Figure 6:**
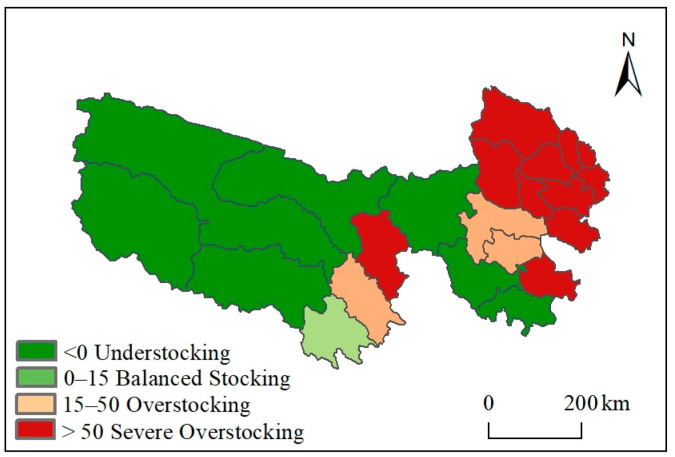
Grass and livestock balance under artificial supplementary feeding conditions.

From a spatial perspective (Figure 6), under an artificial supplementary feeding scenario, the grass–livestock balance in the TRHR shows uneven distribution. Overstocking occurs in the northeastern and central areas, such as Yushu and Chengduo, while the western area is understocked. Specifically, seven counties, accounting for 65.7% of the area, were understocked; one county was balanced, accounting for 3.2%; three counties were overstocked, accounting for 9.1%; and eleven counties were severely overstocked, accounting for 21.9%. Overall, artificial supplementary feeding alleviated the conflict between grass and livestock in the TRHR, with nearly 69% of the area experiencing understocking and 31% experiencing overstocking.

#### 3.4.3. The Grass–Livestock Balance Under Nutrient Carrying Capacity (CC)

Crude protein content is a critical nutritional indicator of forage quality, directly impacting pasture quality and determining the nutritional CC of pastures. Table 3 presents a comprehensive overview of the grass–livestock balance and overgrazing rate of edible forage grasses in the TRHR, considering distinct nutritional carrying capacities at various temporal points. The results showed that from maintaining basic metabolism to maintaining standard sheep body weight unchanged, the grass–livestock balance and overgrazing rate decreased by approximately 4.26%. The grass–livestock balance and overgrazing rate decreased by approximately 6.67% from maintaining the standard sheep weight unchanged to the situation where the standard sheep weight increased by 100 g per day. The above results indicate that under different crude protein requirements, the grass–livestock balance and overgrazing rate in the TRHR are both less than zero, indicating an understocking state, with little variation.

## 4. Discussion

### 4.1. Spatial and Temporal Distribution of Grass Yield and Supply–Demand Contradictions in the TRHR

In terms of spatial scale, the grass yield in the TRHR decreased from the southeast to the northwest from 2000 to 2023. This spatial distribution pattern aligns closely with the laws governing the distribution of water and heat in high-altitude areas. Furthermore, existing research has demonstrated that altitude, temperature, and precipitation significantly affect vegetation and grass yield [33]. The central and southeastern parts of the TRHR have higher grass yields, primarily due to the low altitude and favorable water and heat conditions, which provide optimal natural conditions for vegetation growth. Conversely, the grass yield in the western region is comparatively low due to the presence of extensive desert areas and vegetation-free regions, resulting in sparse vegetation. In addition, the region experiences high altitudes, low temperatures, and low precipitation. From the perspective of time scale and change trends, the grass yield (unit yield) of the TRHR showed a fluctuating upward trend, with more than 83.26% of the area demonstrating a significant increase. This is largely related to the implementation of multiple ecological conservation initiatives in the TRHR by national and Qinghai provincial governments since 2000. These projects include grazing reduction and grassland restoration; black soil restoration; fencing; enclosure; and rodent control [34]. Some studies have shown that these ecological engineering projects have positively impacted the restoration of regional vegetation [35], effectively slowing and even reversing grassland degradation in some areas [36]. As these projects continue to be implemented, grasslands are experiencing restorative growth while improving continuously within their ecological environment [35].

However, this study shows that although grass yield increased in the TRHR, the growth in the CC of natural grasslands was insufficient to meet actual livestock demands. A significant gap in the supply of forage remained. These findings are consistent with other studies, which also found that despite the implementation of strict grass–livestock balance policies and ecological restoration projects spanning several years in the TRHR, natural grasslands remain overgrazed, and grazing pressure exhibits significant spatial heterogeneity [37,38]. In fact, despite the increase in grass yield, rapid population growth and socioeconomic development have led to an increase in the demand for livestock products, such as meat, eggs, and milk [39]. Relevant studies indicate that the consumption of livestock products will continue to grow at a relatively fast pace over the next 20 years [40]. However, livestock products are highly dependent on grassland resources, which can easily lead to overgrazing and a subsequent decline in grassland productivity. This means that if there is excessive reliance on natural grasslands to meet growing demand for livestock products and economic development requirements, achieving a balance between grass supply and demand will remain a major challenge, and the conflict between people, grass, and livestock will continue.

### 4.2. The Balance of Grass and Livestock in the TRHR and the Impact of Artificial Supplementary Feeding on It

**Figure 7:**
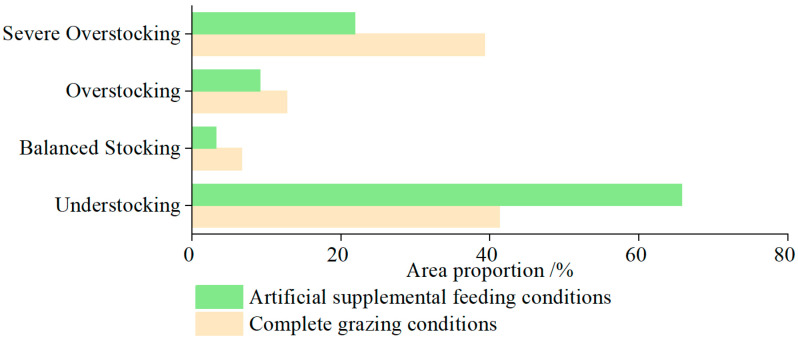
Percentage of area in grass and livestock balance under different scenarios.

Previous studies on calculating the grass–livestock balance have primarily focused on the yield of natural grasslands, with limited consideration of how artificial supplementation and forage quality impact the balance. Therefore, this study thoroughly analyzed the grass–livestock balance in the TRHR under various scenarios. As shown in Figure 7, the results indicate that under complete grazing conditions, over 52% of the TRHR was overstocked, while 48% was not. Under artificial supplementary feeding conditions, nearly 69% of the region was not overstocked, while 31% was. Under nutrient CC conditions, both the grass–livestock balance and overgrazing rate were less than zero under different crude protein requirements, indicating an understocked state. These results suggest that the TRHR has a high nutrient CC for grasslands. When the intensity of natural grassland grazing remained unchanged, implementing artificial supplementary feeding measures reduced the area of overgrazed regions by 21%. This demonstrates that artificial supplementary feeding can effectively address the disparity between forage supply and demand, serving as an essential strategy for advancing the sustainable development of livestock husbandry.

In regions with advanced livestock husbandry (40–50° mid-latitudes), particularly in countries such as Western Europe, New Zealand, and the United States, which are referred to as “golden pastures”, the level of livestock husbandry development far exceeds that of other countries. Wang et al. [41] have shown that in these economically developed regions, the positive ecological effects of human activities on grasslands are more pronounced. Specifically, among the grassland management measures adopted in developed regions, the most notable difference was found in the utilization of artificial pasture. In these developed regions, the proportion of artificial pasture is relatively high, with New Zealand at 67.11%, Western Europe at 40%, and the United States at 12.64%. This indicates that artificial pasture plays an extremely important role in livestock husbandry, and their planting scale and production levels serve as key indicators of a country’s level of livestock husbandry modernization [42].

However, due to long-term extensive production methods in China, as well as the increasing demand for livestock breeding with the socio-economic development in pastoral areas, the grass yield of natural grasslands is far from sufficient to meet the needs of livestock breeding [38]. Therefore, pastoral areas in China have gradually exhibited a dualistic characteristic of natural and artificial pastures [43]. Nevertheless, artificial pastures in China cover only 3% of natural grasslands, and their yield and quality are lower than those in developed regions. Studies have shown that the yield per acre of artificial pasture is 10 to 20 times greater than that of natural grassland [44]. Additionally, the forage quality of artificial pasture is superior, with protein production being 20 to 40 times higher [45]. Given this, it is possible to attempt to protect and restore large areas of natural grassland by using small areas of artificial pasture in the production and living areas on the periphery of the TRHR. That is, on land with low grassland utilization (less than 10%, or even 5%) and suitable water and heat conditions, intensive, high-yielding, and efficient artificial pastures can be established to provide the high-quality forage necessary to support sustainable livestock husbandry development [46].

### 4.3. Influencing Factors and Limitations of Grassland Carrying Capacity (CC) Assessment

The forage yield and nutritional quality of natural grasslands vary significantly across different stages and seasons. Research indicates that winter pastures experience much higher grazing pressure than summer pastures. This is primarily due to their proximity to herder settlements and water facilities. The combination of a longer grazing time and higher grazing intensity usually leads to more severe degradation of winter pastures than of summer pastures [47]. Furthermore, relevant studies have shown that the CC for forage yield and crude protein is lower in the cold season than in the warm season. This is specifically manifested as the peak growth period > the regreening period > the withering period [6,23]. To prevent weight loss in livestock, supplementary feeding should be carried out in autumn and winter to maximize production efficiency and sustainably utilize grasslands.

Another study indicates that there were significant changes in grass production and the capacity for livestock grazing occurred prior to and following the initiation of the ecological project in the TRHR [18,37]. Among them, the average grass yield of the grassland increased by 30.31% compared to before the implementation of the project. After the implementation of the reduction in livestock measures, the average CC index decreased by 36.1% compared to before. At the same time, the pressure from grazing on winter pastures diminished progressively as a result of a decrease in livestock numbers and a shorter grazing season following the ecological project’s implementation. Therefore, the assessment of grassland CC needs to consider not only the dynamic changes in different periods but also the influence of seasonal factors. This includes fully considering the division of pastures in different seasons and the number of grazing days in each season.

Additionally, the supply of forage grasses in natural grasslands is affected by wild herbivores, natural disasters, climate change, and plant invasions [9,38,47,48]. Therefore, future assessments of grassland CC should consider multiple factors. On one hand, the impact of the feeding behavior of wild herbivores, natural disasters, climate change, and plant invasions on the grasslands’ CC needs to be thoroughly evaluated. On the other hand, a comprehensive and systematic evaluation should be conducted based on factors such as grass yield, livestock output and reproduction rates, crude protein yield, and seasonal wild animal migration to enhance the evaluation’s scientific and rational basis.

Although this study considered the uninhabited Hoh Xil region and assigned it a value of zero livestock in the spatial map, certain limitations remain. Future research should establish a more comprehensive grassland CC evaluation system by integrating multi-source data and field survey materials. This system should systematically consider the types of excluded lands, such as those with steep slopes, glacier snow cover, and other non-grazing areas, thereby significantly improving the spatial resolution and reliability of grassland CC assessments.

### 4.4. Recommendations for Improving Grassland Management

To effectively alleviate the temporal and spatial contradiction between grass and livestock, implementing scientific and reasonable grassland management is the key to achieving the prosperity of livestock husbandry and the sustainable utilization of grasslands. The TRHR has reduced grassland pressure through strict ecological protection policies and ecological compensation mechanisms to obtain ecological and economic benefits. However, results show that overgrazing still exists in some areas of the region, and that the expected goals have not been achieved. The following measures could be implemented in the future to improve grassland management.

The establishment of artificial pastures is an important measure for developing an intensive grassland livestock husbandry, implementing ecological restoration and reconstruction, and achieving sustainable development strategies [49]. This approach has demonstrated advantages in various facets of grassland use and the management of livestock [46]. The implementation of artificial pastures can greatly enhance forage production and the capacity for livestock, reduce the ecological stress induced by grazing, and help to even out seasonal discrepancies in feed availability. Therefore, it may be worthwhile to explore cultivating artificial pasture grass in the peripheral areas of the TRHR in the future. This grass could be used for silage, green fodder, semi-dry silage, hay production, or grazing. At the same time, artificial pastures usually require advanced technology that has already been used and proven effective in developed regions [49,50]. These technologies allow for real-time monitoring of livestock, forage growth, and health conditions. This enhances the quantity and quality of forage and livestock products. Additionally, human intervention makes it possible to obtain more and higher-quality forage and mitigate the adverse effects of meteorological disasters and other unfavorable factors. Additionally, as the direct managers and users of grasslands, herders should be trained to use new technologies, enabling them to effectively manage their pastures. Furthermore, it is essential to systematically quantify the economic costs (such as feed, transportation, and labor) and ecological risks (including nutrient imbalance and the invasion of invasive species) associated with manual supplementary feeding. These factors should be integrated with the effectiveness of grassland restoration and the increase in livestock production within a cost–benefit framework to optimize pasture management policies.

One of the key factors contributing to overgrazing is the ineffective implementation of the grass–livestock balance policy. Therefore, strict regulation is essential, as are effective, feasible incentive and disincentive policies, and community participation mechanisms. To address deficiencies in the livestock product distribution system and market, we can establish professional livestock cooperatives, promote large-scale and industrialized operations, and integrate production factors such as capital, technology, and labor. We can optimize the ratio of cattle to sheep and the timing of livestock sales. We should reduce the number of grazing livestock in overgrazed areas, increase investment in artificial supplementary feeding during winter months, and lower the utilization rate of grassland grazing to keep it within a reasonable stocking capacity range. Through these measures, a reasonable allocation and coordinated development of grassland production and ecological functions can be achieved, thereby bringing more economic benefits to livestock husbandry.

Some developed regions have extensive experience in responding to natural disasters and managing grasslands. For example, governments can implement insurance systems that provide herders with economic compensation based on the type and extent of the disaster [36]. Furthermore, certain regions have the potential to advance studies in ecology and promote grassland tourism, increasing the visibility of local livestock products while raising public consciousness about environmental protection.

## 5. Conclusions

Not only is the sustainable development of livestock husbandry crucial to regional ecology, but a sustainable supply of livestock products and healthy livestock husbandry are also important to human well-being. To achieve this, effective management measures need to be implemented to prevent grassland degradation. Among them, how to effectively analyze the grassland CC and the grass–livestock balance situation in different scenarios from multiple dimensions is the key to solving the problem. Therefore, this study used MODIS NPP data to calculate the grass and crude protein yields of grasslands. Then, we calculated the actual and theoretical carrying capacities by livestock and artificial supplementary feed data. Based on this, we analyzed the CC of grasslands and changes in the grass–livestock balance under different scenarios in the TRHR. The results indicated that, despite increases in grass yield and theoretical livestock CC under strict ecological protection measures, the actual livestock CC in the TRHR has increased at a rate exceeding the theoretical capacity. The increase in natural grass yield was not sufficient to meet the actual needs of livestock. The risk of grassland degradation still existed, and ecological pressure remained high. In contrast, although artificial supplementary feeding effectively alleviated grass–livestock conflicts in some areas, overgrazing still occurred.

In fact, regions with developed livestock husbandry primarily benefit economically and ecologically due to their effective utilization and management of grasslands. This includes cultivating artificial pastures, using advanced technology, and implementing other supporting measures. Therefore, drawing on advanced grassland management experiences and concepts from domestic and international sources will promote the TRHR’s coordinated economic and ecological development, as well as harmony between people, grass, and livestock. This will ensure the region’s grasslands are used sustainably.

## Figures and Tables

**Table 1:** Evaluation of grass–livestock balance and overgrazing rate grade classification grading.

Classification	Overgrazing Rate
Understocking	B < 0
Balanced Stocking	0 ≤ B < 15%
Overstocking	15% ≤ B < 50%
Severe Overstocking	B ≥ 50

**Table 2:** Crude protein yield and theoretical carrying capacity (CC) of crude protein at different time periods.

Period	Crude Protein Yield g/m^−2^	Maintenance of Basic Metabolism SU/hm^−2^	Maintenance of 45 kg Body Weight SU/hm^−2^	Standard Sheep Body Weight Gain 100 g per Day SU/hm^−2^
2000	2.54	12.90	7.40	4.58
2005	2.78	14.11	8.09	5.00
2010	3.21	16.32	9.37	5.79
2015	2.67	13.56	7.78	4.81
2020	3.11	15.79	9.06	5.60
2023	3.02	15.36	8.81	5.45

**Table 3:** Grass–livestock balance and overgrazing rate at different stages under nutrient carrying capacity (CC).

Grass–Livestock Balance and Overgrazing Rate	2000	2005	2010	2015	2020	2023
Maintenance of basic metabolism	−0.94	−0.95	−0.95	−0.94	−0.95	−0.95
Maintenance of 45 kg body weight	−0.90	−0.91	−0.92	−0.90	−0.91	−0.90
Standard sheep body weight gain 100 g per day	−0.84	−0.85	−0.86	−0.83	−0.85	−0.85
Maintenance of basic metabolism	−0.94	−0.95	−0.95	−0.94	−0.95	−0.95

## Data Availability

The data utilized in this study are detailed in the Materials and Methods section.

## Abbreviations

The following abbreviations are used in this manuscript:

TRHR	Three River Headwaters Region
CC	carrying capacity
SU	sheep units
NPP	Net Primary Productivity
MK	Mann-Kendall

## References

[1] Ni J. (2004). Estimating Net Primary Productivity of Grasslands from Field Biomass Measurements in Temperate Northern China. Plant Ecol..

[2] Ali I., Cawkwell F., Dwyer E., Barrett B., Green S. (2016). Satellite Remote Sensing of Grasslands: From Observation to Management. J. Plant Ecol..

[3] Zhao Y.J., Song W.J., Wu Z.S., Bai Y.F. (2024). Spatial and temporal analysis of reasonable livestock carrying capacity and ecological carrying capacity of grasslands in pastoral areas of China. Chin. Sci. Bull..

[4] Xu Z., Li X., Zhang L. (2025). Moderate Grazing Intensity and Pika Disturbance Can Enhance Plant Diversity and Soil Nutrient Cycling in the Alpine Grasslands of the Qinghai-Xizang Plateau. Ecol. Front..

[5] Wang Q., Okadera T., Nakayama T., Batkhishig O., Bayarsaikhan U. (2024). Estimation of the Carrying Capacity and Relative Stocking Density of Mongolian Grasslands under Various Adaptation Scenarios. Sci. Total Environ..

[6] Cai Z.Y., Wang J.B., Song P.F., Jiang F., Liang C.B., Zhang J.J., Gao H.M., Zhang T.Z. (2025). The analysis of grazing pressure of ungulates on grassland in Lancang River Source Park, Sanjiangyuan National Park. Acta Ecol. Sin..

[7] Zhang H., Fan J., Wang J., Cao W., Harris W. (2018). Spatial and Temporal Variability of Grassland Yield and Its Response to Climate Change and Anthropogenic Activities on the Tibetan Plateau from 1988 to 2013. Ecol. Indic..

[8] Song W.J., Su J.S., Zhang M.D., Zhao Y.J., Wang Z.W., Jia Y.S., Bai Y.F. (2023). Plant compensatory growth and optimal grazing intensity of grasslands in northern China: A meta-analysis of grazing experiments. Chin. Sci. Bull..

[9] Gao H., Jiang F., Chi X., Li G., Cai Z., Qin W., Zhang J., Wu T., Zhang T. (2020). The Carrying Pressure of Livestock Is Higher than That of Large Wild Herbivores in Yellow River Source Area, China. Ecol. Model..

[10] Xu Q., LI Q., Chen D.d., He F.Q., Chen X., Zhao X.Q., Zhao L. (2017). The spatial-temporal characteristic of land use change in Sanjiangyuan Region and its effect factors. Ecol. Environ. Sci..

[11] Liu J., Xu X., Shao Q. (2008). Grassland Degradation in the “Three-River Headwaters” Region, Qinghai Province. J. Geogr. Sci..

[12] Shu K., Gao X., Qian D., Zhao L., Li Q., Dai L. (2022). Relationship between Biomass and Biodiversity of Degraded Grassland in the Sanjiangyuan Region of Qinghai–Tibet Plateau. Diversity.

[13] Tian D., Xie Y., Barnosky A.D., Wei F. (2019). Defining the Balance Point between Conservation and Development. Conserv. Biol..

[14] Ding L., Yan Q., Liu P., Yang Q., Henkin Z., Degen A.A. (2024). Livestock Turnover and Dynamic Livestock Carrying Capacity Are Crucial Factors for Alpine Grassland Management: The Qinghai-Tibetan Plateau as a Case Study. J. Environ. Manag..

[15] Freeland W.J., Choquenot D. (1990). Determinants of Herbivore Carrying Capacity: Plants, Nutrients, and Equus Asinus in Northern Australia. Ecology.

[16] Bai Y., Zhou S., Wu J., Zeng H., Luo B., Huang M., Qi L., Li W., Shrestha M., Degen A.A. (2025). Estimation of Crude Protein Content in Revegetated Alpine Grassland Using Hyperspectral Data. Remote Sens..

[17] Qi H., Chen A., Yang X., Xing X. (2025). Estimation of Crude Protein Content in Natural Pasture Grass Using Unmanned Aerial Vehicle Hyperspectral Data. Comput. Electron. Agric..

[18] Zhang L.X., Fan J.W., Shao Q.Q., Tang F.P., Zhang H.y., Li Y.Z. (2014). Changes in grassland yield and grazing pressure in the Three Rivers headwater region before and after the implementation of the eco-restoration project. Acta Pratacult. Sin..

[19] Wang S.X., Xu Z.R., Qiao T., Zhang B., Wei Z.Q., Yang X.M. (2023). Intensity of competition for forage between livestock and wild herbivores based on grassland carrying capacity. Prog. Geogr..

[20] Wei Q., Zhou B., Wang W. (2025). Qinghai Province (Tibetan Plateau): Quantifying the Influence of Climate Change and Human Activities on Vegetation Net Primary Productivity and Livestock Carrying Capacity Growth Potential. Biology.

[21] Zhang X., Ning J. (2023). Patterns, Trends, and Causes of Vegetation Change in the Three Rivers Headwaters Region. Land.

[22] Zhao X.Q. (2021). Current Status, Changes, and Management of Ecosystems in Sanjiangyuan National Park.

[23] He F.Q., Chen D.D., Li Q., Huo L.L., Zhao L., Li C.L., Chen X. (2021). Temporal and Spatial Patterns of Herbage and Nutrient Carrying Capacity of Alpine Grassland of Sanjiangyuan. Acta Agrest. Sin..

[24] Guo B., Zang W., Han B., Chen S., Liu Y., Yang X., He T., Chen X., Liu C., Gong R. (2020). Spatial and Temporal Change Patterns of Net Primary Productivity and Its Response to Climate Change in the Qinghai-Tibet Plateau of China from 2000 to 2015. J. Arid Land.

[25] Lu X., Wang J.L., Kang H.J., Zhao Q., Han X.H., Wang Y.J. (2017). Spatio-temporal Changes of Grassland Production Based on MODIS NPP in the Three-River Source Region from 2006 to 2015. J. Nat. Resour..

[26] Wang C.Y., Wang J.B., Zhang F.W., Li Y.N., Li H.Q., Yang Y.S., Luo F.L. (2022). Climate resource utilization rate and livestock-carrying capacity of grasslands in the Three River Headwaters region over the past 40 years. Pratacultural Sci..

[27] Mo X.G., Liu W., Meng C.C., Hu S., Liu S.X., Lin Z.H. (2021). Variations of forage yield and forage⁃livestock balance in grasslands over the Tibetan Plateau, China. Chin. J. Appl. Ecol..

[28] Wang Q., Wu Y.C., Chen K.L., Zhang X., Zhang L.L., Ding J.X. (2019). Estimating grassland yield and carrying capacity in Qinghai Lake Basin based on MODIS NPP data. Ecol. Sci..

[29] Fan J.W., Shao Q.Q., Wang J.B., Chen Z.Q., Zhong H.P. (2011). An Analysis of Temporal-spatial Dynamics of Grazing Pressure on Grassland in Three Rivers Headwater Region. Chin. J. Grassland.

[30] Xia X.S., Ma G.X., Che H.Y., Pan Y.Z., Huang Y.S., Li H.D. (2024). Estimation and change analysis of forage-livestock balance based on cold and warm season grazing region division in Yushu Prefecture, China. Pratacultural Sci..

[31] Qian Q., Zhang X.J., Wang J.B., Ye H., Li Y.N., Zhang Z.J. (2021). TheSpatio-temporalPatternofGrazingPressureintheThree-RiverHeadwaters in Qinghai Province from 2005 to 2017. Acta Agrest. Sin..

[32] Hao L.Z., Liu S.J., Wu K.X., Zhao Y.P., Zhang X.W. (2011). Study on the Evaluation of Grass Nutrition and Carrying Capacity in Alpine Grassland of Kobresia hastily in Maduo County. Chin. J. Grassland.

[33] Yuan X., Guo B., Lu M. (2023). The Responses of Vegetation NPP Dynamics to the Influences of Climate–Human Factors on Qinghai–Tibet Plateau from 2000 to 2020. Remote Sens..

[34] Han Z., Song W., Deng X., Xu X. (2018). Grassland Ecosystem Responses to Climate Change and Human Activities within the Three-River Headwaters Region of China. Sci. Rep..

[35] Zhang X., Jin X. (2021). Vegetation Dynamics and Responses to Climate Change and Anthropogenic Activities in the Three-River Headwaters Region, China. Ecol. Indic..

[36] Cai H., Yang X., Xu X. (2015). Human-Induced Grassland Degradation/Restoration in the Central Tibetan Plateau: The Effects of Ecological Protection and Restoration Projects. Ecol. Eng..

[37] Zhang L., Fan J., Zhou D., Zhang H. (2017). Ecological Protection and Restoration Program Reduced Grazing Pressure in the Three-River Headwaters Region, China. Rangel. Ecol. Manag..

[38] Yang T., Dong J., Huang L., Li Y., Yan H., Zhai J., Wang J., Jin Z., Zhang G. (2023). A Large Forage Gap in Forage Availability in Traditional Pastoral Regions in China. Fundam. Res..

[39] Zhao H., Chang J., Havlík P., van Dijk M., Valin H., Janssens C., Ma L., Bai Z., Herrero M., Smith P. (2021). China’s Future Food Demand and Its Implications for Trade and Environment. Nat. Sustain..

[40] Dong W., Wang X., Yang J. (2015). Future Perspective of China’s Feed Demand and Supply during Its Fast Transition Period of Food Consumption. J. Integr. Agric..

[41] Wang B., Yan H., Zhang Q. (2022). Reciprocity of Grassland Conservation and Pastoralist Livelihoods: Evidence from Comparison between Developed and Developing Regions. Ecol. Indic..

[42] Zhao Z., Bai Y., Deng X., Chen J., Hou J., Li Z. (2020). Changes in Livestock Grazing Efficiency Incorporating Grassland Productivity: The Case of Hulun Buir, China. Land.

[43] Lu H.Y., Wang J., Li H.P., Zheng H.X., Miao P. (2023). Calculation method and application of the tradeoff on water, land, forage, and livestock in family pasture considering forage quality. Trans. Chin. Soc. Agric. Eng..

[44] Fang J.Y., Bai Y.F., Li L.H., Jiang G.M., Huang J.H., Huang Z.Y., Zhang W.H., Gao S.Q. (2016). Scientific basis and practical ways for sustainable development of China’s pasture regions. Chin. Sci. Bull..

[45] Zhou L., Wang L., Zhao B.P., Mi J.Z., Wang F.W., Liu J.H., Zang D., Liu Y.K. (2021). Effect of different sowing date and cutting time on yield and quality of forage oat in agro-pastoral ecotone of northern China. Acta Agrest. Sin..

[46] Fang J.Y., Pan Q.M., Gao S.Q., Jing H.Z., Zhang W.H. (2016). “Small vs. Large Area” Principle: Protecting and restoring a large area of natural grassland by establishing a small area of cultivated pasture. Pratacultural Sci..

[47] Zhang J., Zhang L., Liu W., Qi Y., Wo X. (2014). Livestock-Carrying Capacity and Overgrazing Status of Alpine Grassland in the Three-River Headwaters Region, China. J. Geogr. Sci..

[48] Bardgett R.D., Bullock J.M., Lavorel S., Manning P., Schaffner U., Ostle N., Chomel M., Durigan G., Fry E.L., Johnson D. (2021). Combatting Global Grassland Degradation. Nat. Rev. Earth Environ..

[49] Stampa E., Zander K., Hamm U. (2020). Insights into German Consumers’ Perceptions of Virtual Fencing in Grassland-Based Beef and Dairy Systems: Recommendations for Communication. Animals.

[50] Higgins S., Schellberg J., Bailey J.S. (2019). Improving Productivity and Increasing the Efficiency of Soil Nutrient Management on Grassland Farms in the UK and Ireland Using Precision Agriculture Technology. Eur. J. Agron..

